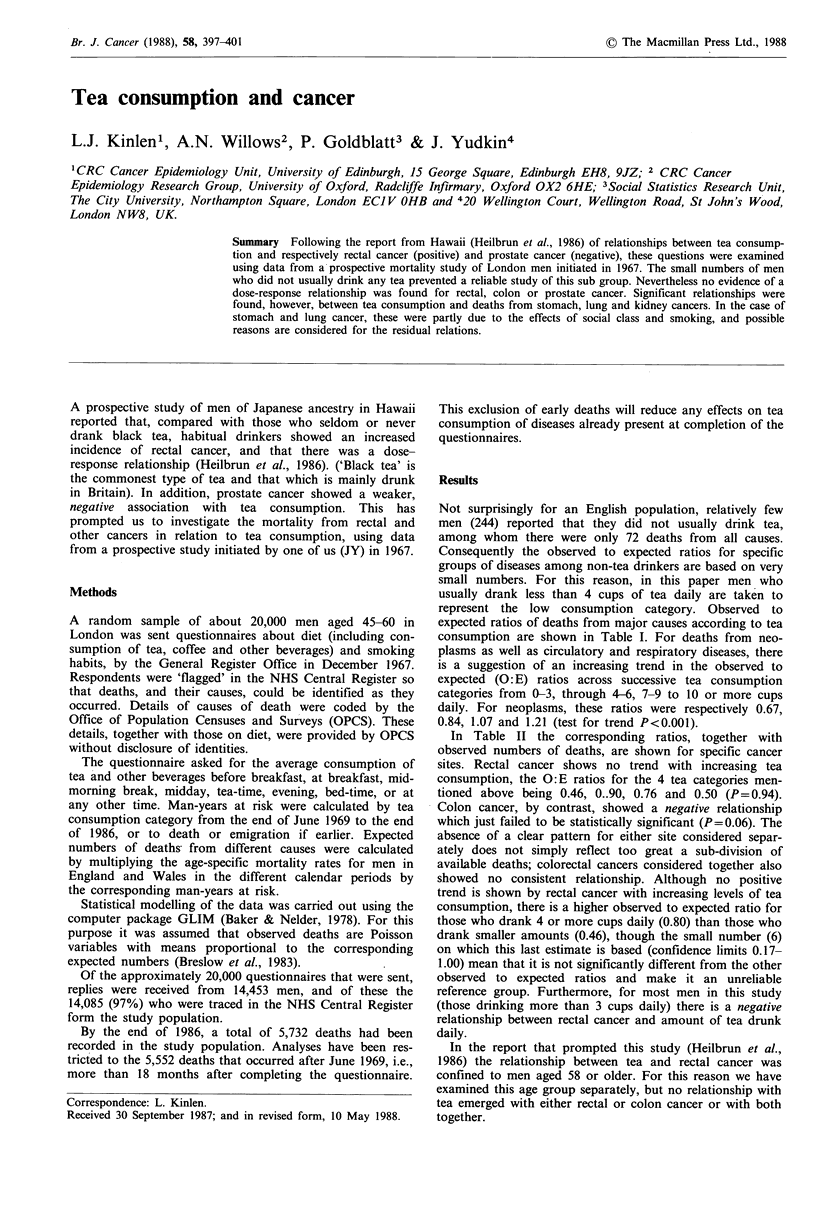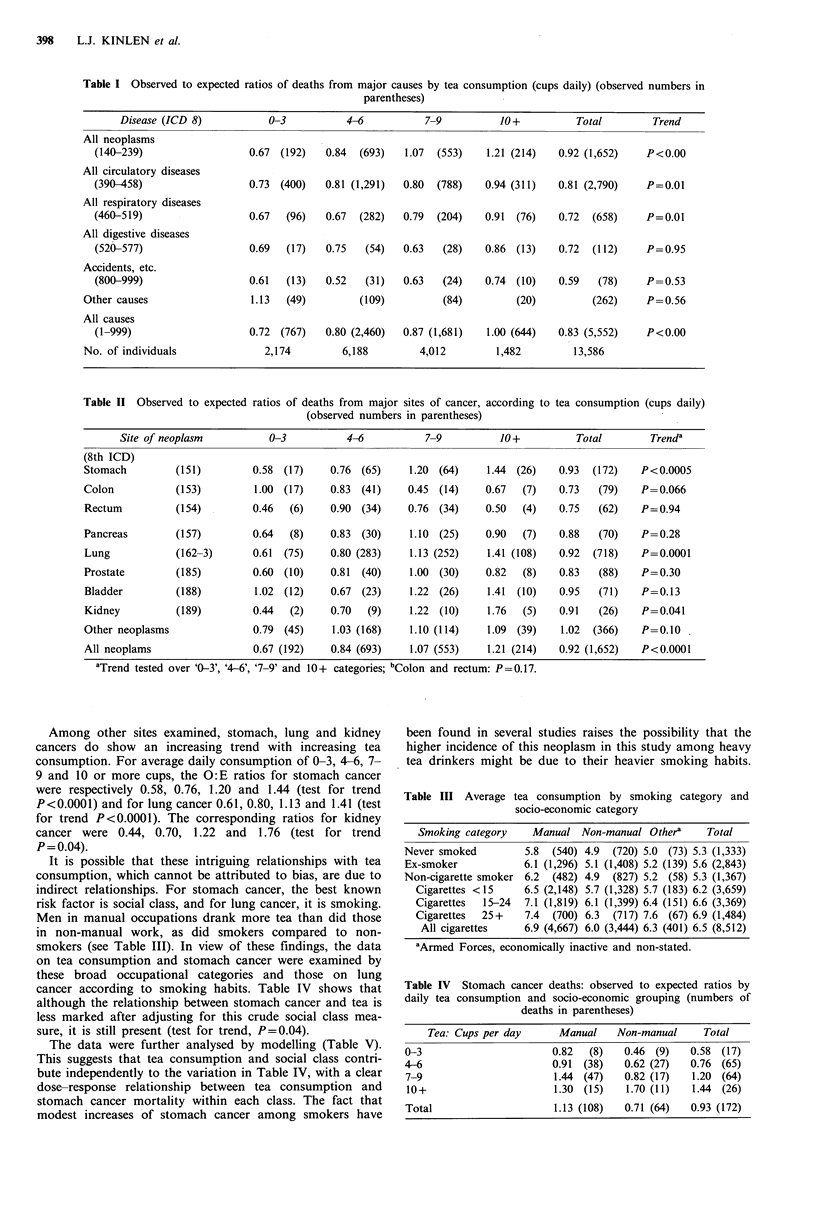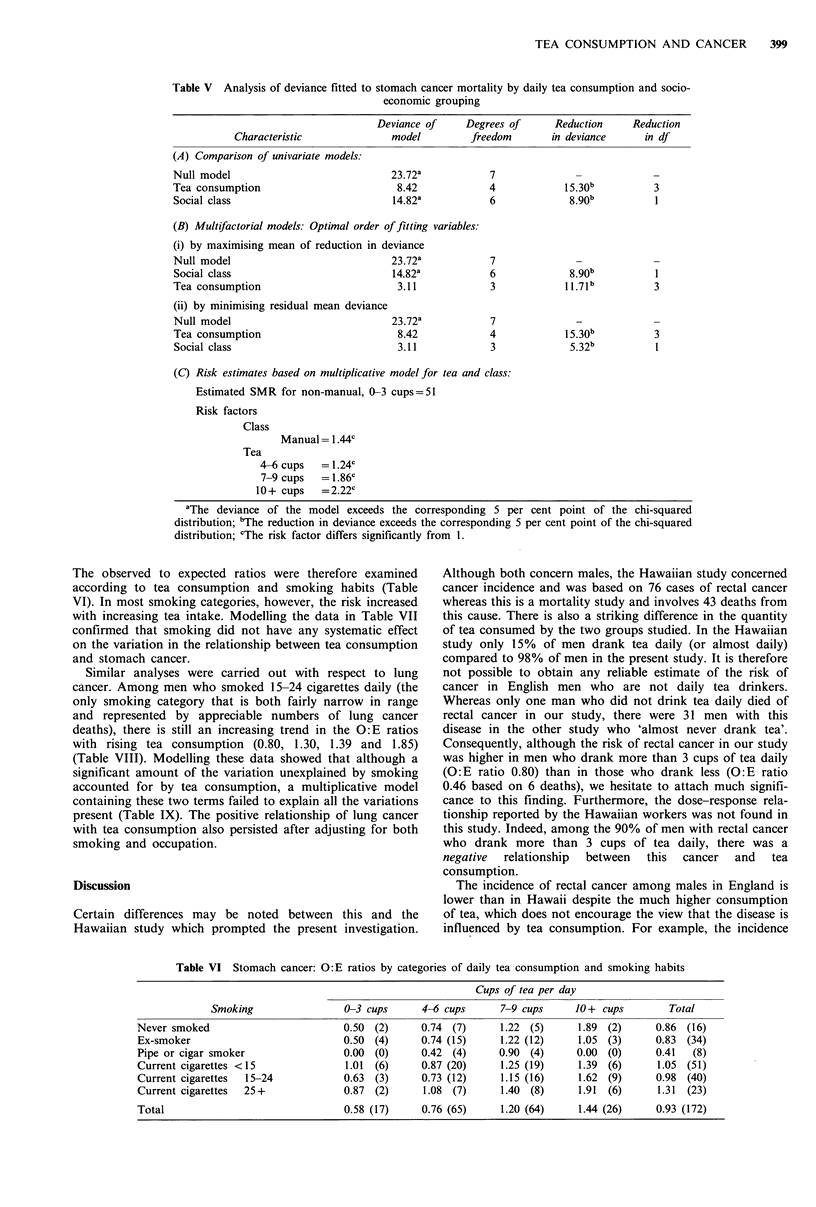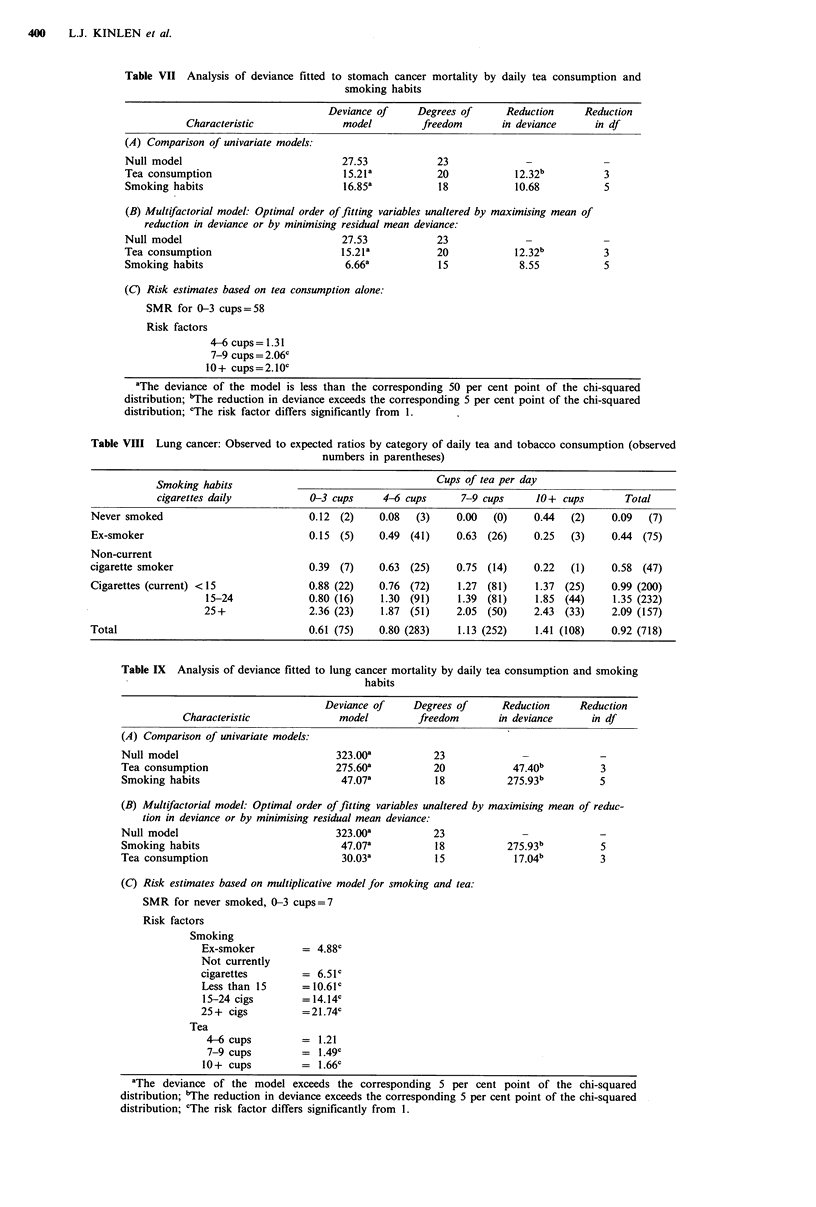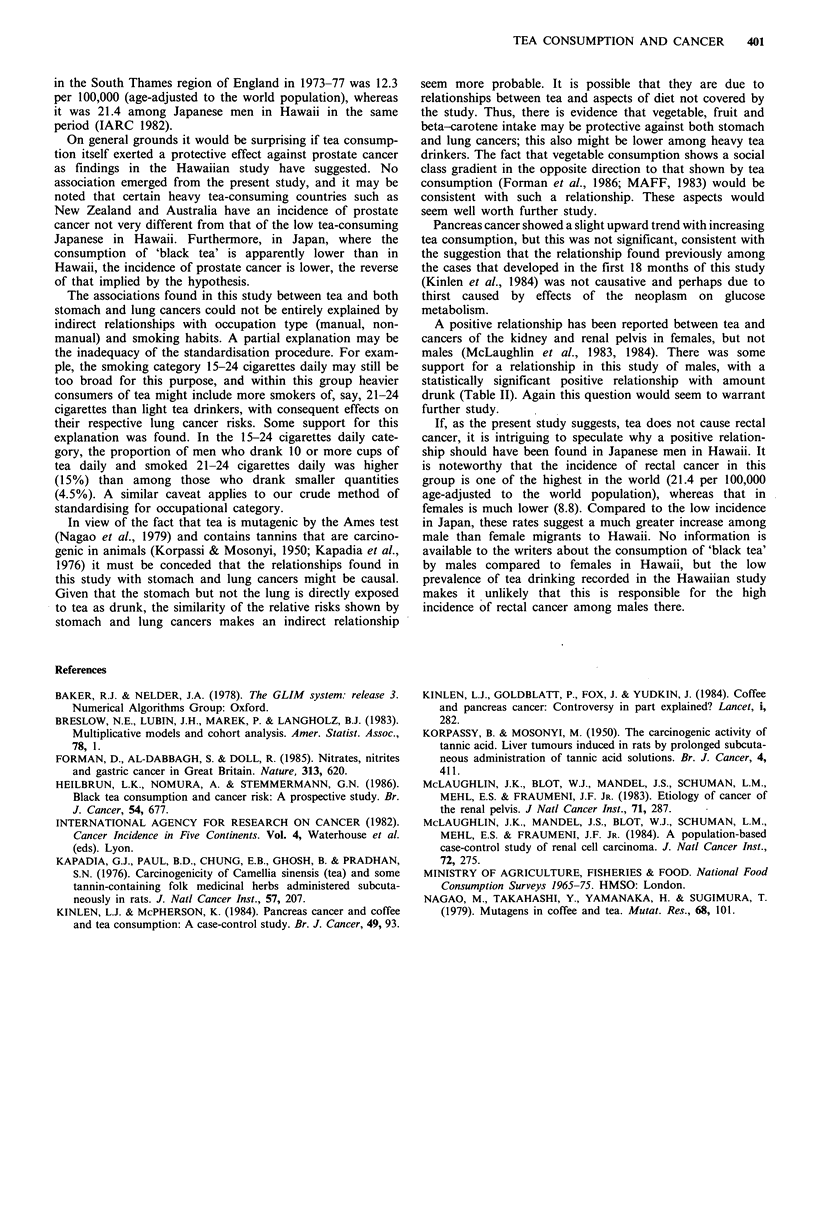# Tea consumption and cancer.

**DOI:** 10.1038/bjc.1988.227

**Published:** 1988-09

**Authors:** L. J. Kinlen, A. N. Willows, P. Goldblatt, J. Yudkin

**Affiliations:** CRC Cancer Epidemiology Unit, University of Edinburgh, UK.

## Abstract

Following the report from Hawaii (Heilbrun et al., 1986) of relationships between tea consumption and respectively rectal cancer (positive) and prostate cancer (negative), these questions were examined using data from a prospective mortality study of London men initiated in 1967. The small numbers of men who did not usually drink any tea prevented a reliable study of this sub group. Nevertheless no evidence of a dose-response relationship was found for rectal, colon or prostate cancer. Significant relationships were found, however, between tea consumption and deaths from stomach, lung and kidney cancers. In the case of stomach and lung cancer, these were partly due to the effects of social class and smoking, and possible reasons are considered for the residual relations.


 © The Macmillan Press Ltd., 1988

### Tea consumption and cancer

L.J. Kinlen[1], A.N. Willows[2], P. Goldblatt[3] & J. Yudkin[4]

[1]*CRC Cancer Epidemiology Unit, University of Edinburgh, 15 George Square, Edinburgh EH8, 9JZ;* [2] *CRC Cancer Epidemiology Research Group, University of Oxford, Radcliffe Infirmary, Oxford OX2 6HE;* [3]*Social Statistics Research Unit, The City University, Northampton Square, London EC1V 0HB and* [4]*20 Wellington Court, Wellington Road, St John's Wood, London NW8, UK.*

**Summary** Following the report from Hawaii (Heilbrun *et al.*, 1986) of relationships between tea consumption and respectively rectal cancer (positive) and prostate cancer (negative), these questions were examined using data from a prospective mortality study of London men initiated in 1967. The small numbers of men who did not usually drink any tea prevented a reliable study of this sub group. Nevertheless no evidence of a dose-response relationship was found for rectal, colon or prostate cancer. Significant relationships were found, however, between tea consumption and deaths from stomach, lung and kidney cancers. In the case of stomach and lung cancer, these were partly due to the effects of social class and smoking, and possible reasons are considered for the residual relations.

A prospective study of men of Japanese ancestry in Hawaii reported that, compared with those who seldom or never drank black tea, habitual drinkers showed an increased incidence of rectal cancer, and that there was a dose–response relationship (Heilbrun *et al.*, 1986). ('Black tea' is the commonest type of tea and that which is mainly drunk in Britain). In addition, prostate cancer showed a weaker, *negative* association with tea consumption. This has prompted us to investigate the mortality from rectal and other cancers in relation to tea consumption, using data from a prospective study initiated by one of us (JY) in 1967.

### Methods

A random sample of about 20,000 men aged 45–60 in London was sent questionnaires about diet (including consumption of tea, coffee and other beverages) and smoking habits, by the General Register Office in December 1967. Respondents were 'flagged' in the NHS Central Register so that deaths, and their causes, could be identified as they occurred. Details of causes of death were coded by the Office of Population Censuses and Surveys (OPCS). These details, together with those on diet, were provided by OPCS without disclosure of identities.

The questionnaire asked for the average consumption of tea and other beverages before breakfast, at breakfast, mid-morning break, midday, tea-time, evening, bed-time, or at any other time. Man-years at risk were calculated by tea consumption category from the end of June 1969 to the end of 1986, or to death or emigration if earlier. Expected numbers of deaths from different causes were calculated by multiplying the age-specific mortality rates for men in England and Wales in the different calendar periods by the corresponding man-years at risk.

Statistical modelling of the data was carried out using the computer package GLIM (Baker & Nelder, 1978). For this purpose it was assumed that observed deaths are Poisson variables with means proportional to the corresponding expected numbers (Breslow *et al.*, 1983).

Of the approximately 20,000 questionnaires that were sent, replies were received from 14,453 men, and of these the 14,085 (97%) who were traced in the NHS Central Register form the study population.

By the end of 1986, a total of 5,732 deaths had been recorded in the study population. Analyses have been restricted to the 5,552 deaths that occurred after June 1969, i.e., more than 18 months after completing the questionnaire. This exclusion of early deaths will reduce any effects on tea consumption of diseases already present at completion of the questionnaires.

Correspondence: L. Kinlen.
Received 30 September 1987; and in revised form, 10 May 1988.

### Results

Not surprisingly for an English population, relatively few men (244) reported that they did not usually drink tea, among whom there were only 72 deaths from all causes. Consequently the observed to expected ratios for specific groups of diseases among non-tea drinkers are based on very small numbers. For this reason, in this paper men who usually drank less than 4 cups of tea daily are taken to represent the low consumption category. Observed to expected ratios of deaths from major causes according to tea consumption are shown in Table I. For deaths from neoplasms as well as circulatory and respiratory diseases, there is a suggestion of an increasing trend in the observed to expected (O:E) ratios across successive tea consumption categories from 0–3, through 4–6, 7–9 to 10 or more cups daily. For neoplasms, these ratios were respectively 0.67, 0.84, 1.07 and 1.21 (test for trend $P<0.001$).

In Table II the corresponding ratios, together with observed numbers of deaths, are shown for specific cancer sites. Rectal cancer shows no trend with increasing tea consumption, the O:E ratios for the 4 tea categories mentioned above being 0.46, 0..90, 0.76 and 0.50 ($P=0.94$). Colon cancer, by contrast, showed a *negative* relationship which just failed to be statistically significant ($P=0.06$). The absence of a clear pattern for either site considered separately does not simply reflect too great a sub-division of available deaths; colorectal cancers considered together also showed no consistent relationship. Although no positive trend is shown by rectal cancer with increasing levels of tea consumption, there is a higher observed to expected ratio for those who drank 4 or more cups daily (0.80) than those who drank smaller amounts (0.46), though the small number (6) on which this last estimate is based (confidence limits 0.17–1.00) mean that it is not significantly different from the other observed to expected ratios and make it an unreliable reference group. Furthermore, for most men in this study (those drinking more than 3 cups daily) there is a *negative* relationship between rectal cancer and amount of tea drunk daily.

In the report that prompted this study (Heilbrun *et al.*, 1986) the relationship between tea and rectal cancer was confined to men aged 58 or older. For this reason we have examined this age group separately, but no relationship with tea emerged with either rectal or colon cancer or with both together.

**Table I** Observed to expected ratios of deaths from major causes by tea consumption (cups daily) (observed numbers in parentheses)

| Disease (ICD 8) | 0–3 | 4–6 | 7–9 | 10+ | Total | Trend |
|---|---|---|---|---|---|---|
| All neoplasms (140–239) | 0.67 (192) | 0.84 (693) | 1.07 (553) | 1.21 (214) | 0.92 (1,652) | $P<0.00$ |
| All circulatory diseases (390–458) | 0.73 (400) | 0.81 (1,291) | 0.80 (788) | 0.94 (311) | 0.81 (2,790) | $P=0.01$ |
| All respiratory diseases (460–519) | 0.67 (96) | 0.67 (282) | 0.79 (204) | 0.91 (76) | 0.72 (658) | $P=0.01$ |
| All digestive diseases (520–577) | 0.69 (17) | 0.75 (54) | 0.63 (28) | 0.86 (13) | 0.72 (112) | $P=0.95$ |
| Accidents, etc. (800–999) | 0.61 (13) | 0.52 (31) | 0.63 (24) | 0.74 (10) | 0.59 (78) | $P=0.53$ |
| Other causes | 1.13 (49) | (109) | (84) | (20) | (262) | $P=0.56$ |
| All causes (1–999) | 0.72 (767) | 0.80 (2,460) | 0.87 (1,681) | 1.00 (644) | 0.83 (5,552) | $P<0.00$ |
| No. of individuals | 2,174 | 6,188 | 4,012 | 1,482 | 13,586 | |

**Table II** Observed to expected ratios of deaths from major sites of cancer, according to tea consumption (cups daily) (observed numbers in parentheses)

| Site of neoplasm | | 0–3 | 4–6 | 7–9 | 10+ | Total | Trend[a] |
|---|---|---|---|---|---|---|---|
| (8th ICD) | | | | | | | |
| Stomach | (151) | 0.58 (17) | 0.76 (65) | 1.20 (64) | 1.44 (26) | 0.93 (172) | $P<0.0005$ |
| Colon | (153) | 1.00 (17) | 0.83 (41) | 0.45 (14) | 0.67 (7) | 0.73 (79) | $P=0.066$ |
| Rectum | (154) | 0.46 (6) | 0.90 (34) | 0.76 (34) | 0.50 (4) | 0.75 (62) | $P=0.94$ |
| Pancreas | (157) | 0.64 (8) | 0.83 (30) | 1.10 (25) | 0.90 (7) | 0.88 (70) | $P=0.28$ |
| Lung | (162–3) | 0.61 (75) | 0.80 (283) | 1.13 (252) | 1.41 (108) | 0.92 (718) | $P=0.0001$ |
| Prostate | (185) | 0.60 (10) | 0.81 (40) | 1.00 (30) | 0.82 (8) | 0.83 (88) | $P=0.30$ |
| Bladder | (188) | 1.02 (12) | 0.67 (23) | 1.22 (26) | 1.41 (10) | 0.95 (71) | $P=0.13$ |
| Kidney | (189) | 0.44 (2) | 0.70 (9) | 1.22 (10) | 1.76 (5) | 0.91 (26) | $P=0.041$ |
| Other neoplasms | | 0.79 (45) | 1.03 (168) | 1.10 (114) | 1.09 (39) | 1.02 (366) | $P=0.10$ |
| All neoplams | | 0.67 (192) | 0.84 (693) | 1.07 (553) | 1.21 (214) | 0.92 (1,652) | $P<0.0001$ |

[a]Trend tested over '0–3', '4–6', '7–9' and 10+ categories; [b]Colon and rectum: $P=0.17$.

Among other sites examined, stomach, lung and kidney cancers do show an increasing trend with increasing tea consumption. For average daily consumption of 0–3, 4–6, 7–9 and 10 or more cups, the O:E ratios for stomach cancer were respectively 0.58, 0.76, 1.20 and 1.44 (test for trend $P<0.0001$) and for lung cancer 0.61, 0.80, 1.13 and 1.41 (test for trend $P<0.0001$). The corresponding ratios for kidney cancer were 0.44, 0.70, 1.22 and 1.76 (test for trend $P=0.04$).

It is possible that these intriguing relationships with tea consumption, which cannot be attributed to bias, are due to indirect relationships. For stomach cancer, the best known risk factor is social class, and for lung cancer, it is smoking. Men in manual occupations drank more tea than did those in non-manual work, as did smokers compared to non-smokers (see Table III). In view of these findings, the data on tea consumption and stomach cancer were examined by these broad occupational categories and those on lung cancer according to smoking habits. Table IV shows that although the relationship between stomach cancer and tea is less marked after adjusting for this crude social class measure, it is still present (test for trend, $P=0.04$).

The data were further analysed by modelling (Table V). This suggests that tea consumption and social class contribute independently to the variation in Table IV, with a clear dose–response relationship between tea consumption and stomach cancer mortality within each class. The fact that modest increases of stomach cancer among smokers have been found in several studies raises the possibility that the higher incidence of this neoplasm in this study among heavy tea drinkers might be due to their heavier smoking habits.

**Table III** Average tea consumption by smoking category and socio-economic category

| Smoking category | Manual | Non-manual | Other[a] | Total |
|---|---|---|---|---|
| Never smoked | 5.8 (540) | 4.9 (720) | 5.0 (73) | 5.3 (1,333) |
| Ex-smoker | 6.1 (1,296) | 5.1 (1,408) | 5.2 (139) | 5.6 (2,843) |
| Non-cigarette smoker | 6.2 (482) | 4.9 (827) | 5.2 (58) | 5.3 (1,367) |
| Cigarettes <15 | 6.5 (2,148) | 5.7 (1,328) | 5.7 (183) | 6.2 (3,659) |
| Cigarettes 15–24 | 7.1 (1,819) | 6.1 (1,399) | 6.4 (151) | 6.6 (3,369) |
| Cigarettes 25+ | 7.4 (700) | 6.3 (717) | 7.6 (67) | 6.9 (1,484) |
| All cigarettes | 6.9 (4,667) | 6.0 (3,444) | 6.3 (401) | 6.5 (8,512) |

[a]Armed Forces, economically inactive and non-stated.

**Table IV** Stomach cancer deaths: observed to expected ratios by daily tea consumption and socio-economic grouping (numbers of deaths in parentheses)

| Tea: Cups per day | Manual | Non-manual | Total |
|---|---|---|---|
| 0–3 | 0.82 (8) | 0.46 (9) | 0.58 (17) |
| 4–6 | 0.91 (38) | 0.62 (27) | 0.76 (65) |
| 7–9 | 1.44 (47) | 0.82 (17) | 1.20 (64) |
| 10+ | 1.30 (15) | 1.70 (11) | 1.44 (26) |
| Total | 1.13 (108) | 0.71 (64) | 0.93 (172) |

**Table V** Analysis of deviance fitted to stomach cancer mortality by daily tea consumption and socio-economic grouping

| *Characteristic* | *Deviance of model* | *Degrees of freedom* | *Reduction in deviance* | *Reduction in df* |
|---|---|---|---|---|
| (*A*) *Comparison of univariate models:* | | | | |
| Null model | 23.72[a] | 7 | – | – |
| Tea consumption | 8.42 | 4 | 15.30[b] | 3 |
| Social class | 14.82[a] | 6 | 8.90[b] | 1 |
| (*B*) *Multifactorial models: Optimal order of fitting variables:* | | | | |
| (i) by maximising mean of reduction in deviance | | | | |
| Null model | 23.72[a] | 7 | – | – |
| Social class | 14.82[a] | 6 | 8.90[b] | 1 |
| Tea consumption | 3.11 | 3 | 11.71[b] | 3 |
| (ii) by minimising residual mean deviance | | | | |
| Null model | 23.72[a] | 7 | – | – |
| Tea consumption | 8.42 | 4 | 15.30[b] | 3 |
| Social class | 3.11 | 3 | 5.32[b] | 1 |
| (*C*) *Risk estimates based on multiplicative model for tea and class:* | | | | |
| Estimated SMR for non-manual, 0–3 cups = 51 | | | | |
| Risk factors | | | | |
| Class | | | | |
| Manual = 1.44[c] | | | | |
| Tea | | | | |
| 4–6 cups = 1.24[c] | | | | |
| 7–9 cups = 1.86[c] | | | | |
| 10+ cups = 2.22[c] | | | | |

[a]The deviance of the model exceeds the corresponding 5 per cent point of the chi-squared distribution; [b]The reduction in deviance exceeds the corresponding 5 per cent point of the chi-squared distribution; [c]The risk factor differs significantly from 1.

The observed to expected ratios were therefore examined according to tea consumption and smoking habits (Table VI). In most smoking categories, however, the risk increased with increasing tea intake. Modelling the data in Table VII confirmed that smoking did not have any systematic effect on the variation in the relationship between tea consumption and stomach cancer.

Similar analyses were carried out with respect to lung cancer. Among men who smoked 15–24 cigarettes daily (the only smoking category that is both fairly narrow in range and represented by appreciable numbers of lung cancer deaths), there is still an increasing trend in the O:E ratios with rising tea consumption (0.80, 1.30, 1.39 and 1.85) (Table VIII). Modelling these data showed that although a significant amount of the variation unexplained by smoking accounted for by tea consumption, a multiplicative model containing these two terms failed to explain all the variations present (Table IX). The positive relationship of lung cancer with tea consumption also persisted after adjusting for both smoking and occupation.

### Discussion

Certain differences may be noted between this and the Hawaiian study which prompted the present investigation. Although both concern males, the Hawaiian study concerned cancer incidence and was based on 76 cases of rectal cancer whereas this is a mortality study and involves 43 deaths from this cause. There is also a striking difference in the quantity of tea consumed by the two groups studied. In the Hawaiian study only 15% of men drank tea daily (or almost daily) compared to 98% of men in the present study. It is therefore not possible to obtain any reliable estimate of the risk of cancer in English men who are not daily tea drinkers. Whereas only one man who did not drink tea daily died of rectal cancer in our study, there were 31 men with this disease in the other study who 'almost never drank tea'. Consequently, although the risk of rectal cancer in our study was higher in men who drank more than 3 cups of tea daily (O:E ratio 0.80) than in those who drank less (O:E ratio 0.46 based on 6 deaths), we hesitate to attach much significance to this finding. Furthermore, the dose–response relationship reported by the Hawaiian workers was not found in this study. Indeed, among the 90% of men with rectal cancer who drank more than 3 cups of tea daily, there was a *negative* relationship between this cancer and tea consumption.

The incidence of rectal cancer among males in England is lower than in Hawaii despite the much higher consumption of tea, which does not encourage the view that the disease is influenced by tea consumption. For example, the incidence

**Table VI** Stomach cancer: O:E ratios by categories of daily tea consumption and smoking habits

| | *Cups of tea per day* | | | | |
|---|---|---|---|---|---|
| *Smoking* | *0–3 cups* | *4–6 cups* | *7–9 cups* | *10+ cups* | *Total* |
| Never smoked | 0.50 (2) | 0.74 (7) | 1.22 (5) | 1.89 (2) | 0.86 (16) |
| Ex-smoker | 0.50 (4) | 0.74 (15) | 1.22 (12) | 1.05 (3) | 0.83 (34) |
| Pipe or cigar smoker | 0.00 (0) | 0.42 (4) | 0.90 (4) | 0.00 (0) | 0.41 (8) |
| Current cigarettes <15 | 1.01 (6) | 0.87 (20) | 1.25 (19) | 1.39 (6) | 1.05 (51) |
| Current cigarettes 15–24 | 0.63 (3) | 0.73 (12) | 1.15 (16) | 1.62 (9) | 0.98 (40) |
| Current cigarettes 25+ | 0.87 (2) | 1.08 (7) | 1.40 (8) | 1.91 (6) | 1.31 (23) |
| Total | 0.58 (17) | 0.76 (65) | 1.20 (64) | 1.44 (26) | 0.93 (172) |

**Table VII** Analysis of deviance fitted to stomach cancer mortality by daily tea consumption and smoking habits

| *Characteristic* | *Deviance of model* | *Degrees of freedom* | *Reduction in deviance* | *Reduction in df* |
|---|---|---|---|---|
| (*A*) *Comparison of univariate models:* | | | | |
| Null model | 27.53 | 23 | – | – |
| Tea consumption | 15.21[a] | 20 | 12.32[b] | 3 |
| Smoking habits | 16.85[a] | 18 | 10.68 | 5 |
| (*B*) *Multifactorial model: Optimal order of fitting variables unaltered by maximising mean of reduction in deviance or by minimising residual mean deviance:* | | | | |
| Null model | 27.53 | 23 | – | – |
| Tea consumption | 15.21[a] | 20 | 12.32[b] | 3 |
| Smoking habits | 6.66[a] | 15 | 8.55 | 5 |
| (*C*) *Risk estimates based on tea consumption alone:* | | | | |
| SMR for 0–3 cups = 58 | | | | |
| Risk factors | | | | |
| 4–6 cups = 1.31 | | | | |
| 7–9 cups = 2.06[c] | | | | |
| 10+ cups = 2.10[c] | | | | |

[a]The deviance of the model is less than the corresponding 50 per cent point of the chi-squared distribution; [b]The reduction in deviance exceeds the corresponding 5 per cent point of the chi-squared distribution; [c]The risk factor differs significantly from 1.

**Table VIII** Lung cancer: Observed to expected ratios by category of daily tea and tobacco consumption (observed numbers in parentheses)

| *Smoking habits cigarettes daily* | *Cups of tea per day* | | | | |
|---|---|---|---|---|---|
| | *0–3 cups* | *4–6 cups* | *7–9 cups* | *10+ cups* | *Total* |
| Never smoked | 0.12 (2) | 0.08 (3) | 0.00 (0) | 0.44 (2) | 0.09 (7) |
| Ex-smoker | 0.15 (5) | 0.49 (41) | 0.63 (26) | 0.25 (3) | 0.44 (75) |
| Non-current cigarette smoker | 0.39 (7) | 0.63 (25) | 0.75 (14) | 0.22 (1) | 0.58 (47) |
| Cigarettes (current) <15 | 0.88 (22) | 0.76 (72) | 1.27 (81) | 1.37 (25) | 0.99 (200) |
| 15–24 | 0.80 (16) | 1.30 (91) | 1.39 (81) | 1.85 (44) | 1.35 (232) |
| 25+ | 2.36 (23) | 1.87 (51) | 2.05 (50) | 2.43 (33) | 2.09 (157) |
| Total | 0.61 (75) | 0.80 (283) | 1.13 (252) | 1.41 (108) | 0.92 (718) |

**Table IX** Analysis of deviance fitted to lung cancer mortality by daily tea consumption and smoking habits

| *Characteristic* | *Deviance of model* | *Degrees of freedom* | *Reduction in deviance* | *Reduction in df* |
|---|---|---|---|---|
| (*A*) *Comparison of univariate models:* | | | | |
| Null model | 323.00[a] | 23 | – | – |
| Tea consumption | 275.60[a] | 20 | 47.40[b] | 3 |
| Smoking habits | 47.07[a] | 18 | 275.93[b] | 5 |
| (*B*) *Multifactorial model: Optimal order of fitting variables unaltered by maximising mean of reduction in deviance or by minimising residual mean deviance:* | | | | |
| Null model | 323.00[a] | 23 | – | – |
| Smoking habits | 47.07[a] | 18 | 275.93[b] | 5 |
| Tea consumption | 30.03[a] | 15 | 17.04[b] | 3 |
| (*C*) *Risk estimates based on multiplicative model for smoking and tea:* | | | | |
| SMR for never smoked, 0–3 cups = 7 | | | | |
| Risk factors | | | | |
| Smoking | | | | |
| Ex-smoker = 4.88[c] | | | | |
| Not currently cigarettes = 6.51[c] | | | | |
| Less than 15 = 10.61[c] | | | | |
| 15–24 cigs = 14.14[c] | | | | |
| 25+ cigs = 21.74[c] | | | | |
| Tea | | | | |
| 4–6 cups = 1.21 | | | | |
| 7–9 cups = 1.49[c] | | | | |
| 10+ cups = 1.66[c] | | | | |

[a]The deviance of the model exceeds the corresponding 5 per cent point of the chi-squared distribution; [b]The reduction in deviance exceeds the corresponding 5 per cent point of the chi-squared distribution; [c]The risk factor differs significantly from 1.

in the South Thames region of England in 1973–77 was 12.3 per 100,000 (age-adjusted to the world population), whereas it was 21.4 among Japanese men in Hawaii in the same period (IARC 1982).

On general grounds it would be surprising if tea consumption itself exerted a protective effect against prostate cancer as findings in the Hawaiian study have suggested. No association emerged from the present study, and it may be noted that certain heavy tea-consuming countries such as New Zealand and Australia have an incidence of prostate cancer not very different from that of the low tea-consuming Japanese in Hawaii. Furthermore, in Japan, where the consumption of 'black tea' is apparently lower than in Hawaii, the incidence of prostate cancer is lower, the reverse of that implied by the hypothesis.

The associations found in this study between tea and both stomach and lung cancers could not be entirely explained by indirect relationships with occupation type (manual, non-manual) and smoking habits. A partial explanation may be the inadequacy of the standardisation procedure. For example, the smoking category 15–24 cigarettes daily may still be too broad for this purpose, and within this group heavier consumers of tea might include more smokers of, say, 21–24 cigarettes than light tea drinkers, with consequent effects on their respective lung cancer risks. Some support for this explanation was found. In the 15–24 cigarettes daily category, the proportion of men who drank 10 or more cups of tea daily and smoked 21–24 cigarettes daily was higher (15%) than among those who drank smaller quantities (4.5%). A similar caveat applies to our crude method of standardising for occupational category.

In view of the fact that tea is mutagenic by the Ames test (Nagao *et al.*, 1979) and contains tannins that are carcinogenic in animals (Korpassi & Mosonyi, 1950; Kapadia *et al.*, 1976) it must be conceded that the relationships found in this study with stomach and lung cancers might be causal. Given that the stomach but not the lung is directly exposed to tea as drunk, the similarity of the relative risks shown by stomach and lung cancers makes an indirect relationship seem more probable. It is possible that they are due to relationships between tea and aspects of diet not covered by the study. Thus, there is evidence that vegetable, fruit and beta-carotene intake may be protective against both stomach and lung cancers; this also might be lower among heavy tea drinkers. The fact that vegetable consumption shows a social class gradient in the opposite direction to that shown by tea consumption (Forman *et al.*, 1986; MAFF, 1983) would be consistent with such a relationship. These aspects would seem well worth further study.

Pancreas cancer showed a slight upward trend with increasing tea consumption, but this was not significant, consistent with the suggestion that the relationship found previously among the cases that developed in the first 18 months of this study (Kinlen *et al.*, 1984) was not causative and perhaps due to thirst caused by effects of the neoplasm on glucose metabolism.

A positive relationship has been reported between tea and cancers of the kidney and renal pelvis in females, but not males (McLaughlin *et al.*, 1983, 1984). There was some support for a relationship in this study of males, with a statistically significant positive relationship with amount drunk (Table II). Again this question would seem to warrant further study.

If, as the present study suggests, tea does not cause rectal cancer, it is intriguing to speculate why a positive relationship should have been found in Japanese men in Hawaii. It is noteworthy that the incidence of rectal cancer in this group is one of the highest in the world (21.4 per 100,000 age-adjusted to the world population), whereas that in females is much lower (8.8). Compared to the low incidence in Japan, these rates suggest a much greater increase among male than female migrants to Hawaii. No information is available to the writers about the consumption of 'black tea' by males compared to females in Hawaii, but the low prevalence of tea drinking recorded in the Hawaiian study makes it unlikely that this is responsible for the high incidence of rectal cancer among males there.

### References

BAKER, R.J. & NELDER, J.A. (1978). *The GLIM system: release 3.* Numerical Algorithms Group: Oxford.

BRESLOW, N.E., LUBIN, J.H., MAREK, P. & LANGHOLZ, B.J. (1983). Multiplicative models and cohort analysis. *Amer. Statist. Assoc.*, **78**, 1.

FORMAN, D., AL-DABBAGH, S. & DOLL, R. (1985). Nitrates, nitrites and gastric cancer in Great Britain. *Nature,* **313**, 620.

HEILBRUN, L.K., NOMURA, A. & STEMMERMANN, G.N. (1986). Black tea consumption and cancer risk: A prospective study. *Br. J. Cancer*, **54**, 677.

INTERNATIONAL AGENCY FOR RESEARCH ON CANCER (1982). *Cancer Incidence in Five Continents.* **Vol. 4**, Waterhouse *et al.* (eds). Lyon.

KAPADIA, G.J., PAUL, B.D., CHUNG, E.B., GHOSH, B. & PRADHAN, S.N. (1976). Carcinogenicity of Camellia sinensis (tea) and some tannin-containing folk medicinal herbs administered subcutaneously in rats. *J. Natl Cancer Inst.*, **57**, 207.

KINLEN, L.J. & McPHERSON, K. (1984). Pancreas cancer and coffee and tea consumption: A case-control study. *Br. J. Cancer*, **49**, 93.

KINLEN, L.J., GOLDBLATT, P., FOX, J. & YUDKIN, J. (1984). Coffee and pancreas cancer: Controversy in part explained? *Lancet*, **i**, 282.

KORPASSY, B. & MOSONYI, M. (1950). The carcinogenic activity of tannic acid. Liver tumours induced in rats by prolonged subcutaneous administration of tannic acid solutions. *Br. J. Cancer*, **4**, 411.

McLAUGHLIN, J.K., BLOT, W.J., MANDEL, J.S., SCHUMAN, L.M., MEHL, E.S. & FRAUMENI, J.F. JR. (1983). Etiology of cancer of the renal pelvis. *J Natl Cancer Inst.*, **71**, 287.

McLAUGHLIN, J.K., MANDEL, J.S., BLOT, W.J., SCHUMAN, L.M., MEHL, E.S. & FRAUMENI, J.F. JR. (1984). A population-based case-control study of renal cell carcinoma. *J. Natl Cancer Inst.*, **72**, 275.

MINISTRY OF AGRICULTURE, FISHERIES & FOOD. *National Food Consumption Surveys 1965–75.* HMSO: London.

NAGAO, M., TAKAHASHI, Y., YAMANAKA, H. & SUGIMURA, T. (1979). Mutagens in coffee and tea. *Mutat. Res.*, **68**, 101.